# Imatinib-based therapy in adult Philadelphia chromosome-positive acute lymphoblastic leukemia: A case report and literature review

**DOI:** 10.3892/ol.2015.3539

**Published:** 2015-07-28

**Authors:** LI ZHANG, MENG CHEN, BO FENG, PU KUANG, PENG HE, TING LIU, LING PAN

**Affiliations:** 1 Department of Hematology, West China Hospital, Sichuan University, Chengdu, Sichuan 610041, P.R. China; 2 Third Division of Internal Medicine, District People's Hospital, Qionglai Medical Center Hospital, Qionglai, Sichuan 611530, P.R. China

**Keywords:** osteoporosis, fracture, acute lymphoblastic leukemia, imatinib

## Abstract

Acute lymphoblastic leukemia (ALL) has a rapid onset and rarely occurs with exclusive prodrome of general osteoporosis and vertebral compression fractures. However, Philadelphia chromosome-positive (Ph^+^) ALL has a poor prognosis, even when patients are treated with intensive chemotherapy, and the first-line effective treatment requires further elucidation. The present study focused on a 56-year-old Chinese male patient who initially presented with spontaneous bone fractures and was ultimately diagnosed as Ph^+^ ALL after 6 months, which required to preliminarily exclude a working diagnosis of myeloma. Apart from intensive chemotherapy, the patient successfully completed an imatinib-based regimen and achieved complete remission (CR) 2 weeks later. Subsequently, the patient was subjected to consolidation treatment using the same imatinib regimen combined with interferon-α 2b for 9 courses. In November 2013, the patient had achieved persistent hematological and molecular genetic normality for ~16 months after the initial CR. In conclusion, Ph^+^ ALL must be considered in the differential diagnosis of adults experiencing unexplained bone disease.

## Introduction

Acute lymphoblastic leukemia (ALL) is a neoplasm of precursor cells committed to the B-cell and T-cell lineages involving the bone marrow and blood (1). ALL occasionally presents with primary involvement of nodal sites. Osteoporosis and spontaneous vertebral compression fractures as presentations of ALL have been described mainly in pediatric patients, but rarely in adult patients with ALL (2,3). Philadelphia chromosome-positive (Ph^+^) ALL accounts for 25–30% of adult ALL and its incidence increases with age in adults >40 years old. Prior to the era of tyrosine kinase inhibitor (TKI) treatment, Ph^+^ ALL was considered to be a high-risk subgroup among the ALL population. Current intensive chemotherapy regimes have improved the complete remission (CR) rate, however, long-term overall survival (OS) has not improved. In the TKI era, dramatic changes in therapeutic efficacy have occurred, with markedly improved molecular CR and improved OS rates (4). These changes raise questions as to whether the conventional intensive combined chemotherapy treatment regime should remain the front-line treatment strategy for patients with Ph^+^ ALL.

The present study reported the case of a Ph^+^ ALL patient who was treated with imatinib-based individual therapy, which included conventional standard-dose chemotherapy, rather than intensive combined regimens. Imatinib is a first generation TKI, which specifically targets the adenosine triphosphate binding site of the BCR/ABL kinase domain. The current case was characterized by initial presentation of general osteoporosis and vertebral compression fracture.

## Case report

A 56-year-old previously healthy male was originally referred to the West China Hospital (Sichuan University, Chengdu) in June 2012. The patient presented with a 6-month history of progressive lower back and bilateral rib pain. Initially, the patient experienced sudden onset of sharp lower back pain, and a spine X-ray in the local community clinic indicated slipped disc at L2-3, L3-4 and L4-5 levels with a normal complete blood count and white cell differentiation. Acute pain was usually resolved spontaneously, but pain recurrence was frequent. After 3 months, the perceived intensity of pain became more aggressive, followed by emergence of fatigue and dizziness. A repeated complete blood count indicated a hemoglobin level of 6.5 g/dl [normal range (NR), 13.0–17.5 g/dl] and platelet count of 15,000/µl. A computed tomography (CT) scan revealed large absorption and compression fracture of T8 and L3. Red blood cell transfusion was performed in the local clinic prior to admission to the West China Hospital. Upon physical examination, pallor was observed most prominently at the face, eyelids, lips and palms. No lymphadenopathy or hepatosplenomegaly were identified. The findings of a neurologic examination, including strength testing, were normal, and other examinations were unremarkable.

**Figure 1.**
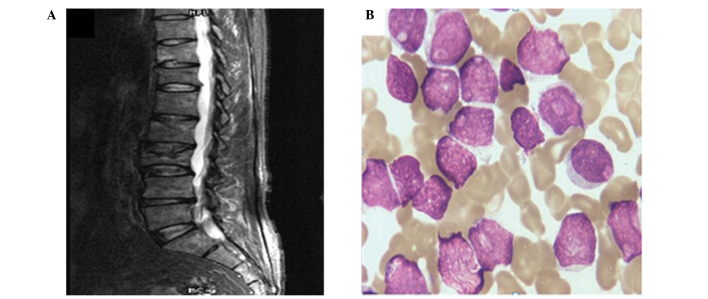
(A) Magnetic resonance imaging revealed impaired physiological curve of thoracolumbar spine, clusters of iso/hypo mixed signals and pathological fracture in T8 and L3. (B) Bone marrow aspiration demonstrated active hyperplasia and a blast cell count of 90.0%.

A complete blood count revealed the following: hemoglobin level, 9.3 g/dl; white blood cell count, 4,780/µl (NR, 3,500–9,500/µl) with 3% blast cells; platelet count, 21,000/µl (NR, 100,000–300,000/µl); lactate dehydrogenase, 1,082 IU/l (NR, 72–182 IU/l); alkaline phosphatase, 6.72 µg/l (NR, 11.4–24.6 µg/l); Type I collagen carboxyl terminal peptide, 0.125 ng/ml (NR, 0.3–0.58 ng/ml); calcium level, 2.16 mmol/l (NR, 2.1–2.7 mmol/l); and inorganic phosphorus level, 0.74 mmol/l (NR, 0.81–1.45 mmol/l). Magnetic resonance imaging (MRI) scans revealed an impaired physiological curve of thoracolumbar spine, clusters of iso/hypo mixed signals and pathological fracture in T8 and L3 with inhomogeneous contrast enhancement (Fig. 1A). A chest CT image demonstrated bone destruction of the sternum, thoracic vertebra, bilateral rib and scapular. In addition, an X-ray showed sheet and irregular low-density lesions on the cranial and maxillofacial bone, pelvis and bilateral proximal femur. Based on this data, a diagnosis of multiple myeloma (MM) was initially made. However, immunofixation electrophoresis (IFE), serum protein electrophoresis (SPE) and urine light chain detection indicated no evidence of monoclonal protein. Furthermore, a bone marrow smear identified active hyperplasia and a blast cell count of 90.0% (Fig. 1B), while the patient was peroxidase-negative. The immunophenotype of the leukemic blasts was analyzed by flow cytometry (FCM) and was as high as the typical phenotype of common-B-cell ALL patients, with CD10^+^, CD19^+^, CD13^+^, CD20^+^, cCD79a^+^, CD34^+^ and HLA-DR^+^. Cytogenetic analysis revealed 46,XY,t(9;22)(q34;q11) [20/20], while a molecular biology examination demonstrated that the patient was *BCR*/*ABL*-positive, as determined by a reverse transcription-polymerase chain reaction method (5). Therefore, a diagnosis of Ph^+^ ALL was ultimately determined according to the Morphology, Immunology, Cytogenetics, Molecular Biology criteria (6).

In addition to administration of 90 mg pamidronate disodium (Bonin; Shenzhen Neptunus Bioengineering Co., Ltd., Shenzhen, China) to reduce bone pain, the patient was treated with the IVD regimen (imatinib, 400 mg/day; vindesine, 4 mg/day, on days 1, 8, 15 and 22; dexamethasone, 10 mg/m^2^/day, on days 1–5, 8–12, 15–19 and 22–26). Hematological CR with rapid resolution of pain was observed 2 weeks after the termination of inductive treatment, which was confirmed by a bone marrow smear and FCM. Meanwhile, the patient continued extramural treatment with imatinib at 400 mg/day. Due to the lack of a matched donor, bone marrow transplantation was not considered for this case. During subsequent consolidation therapy, the CAM (cyclophosphamide, 750 mg/m^2^, days 1 and 8; cytarabine, 100 mg/m^2^, days 1–3 and 8–10; 6-mercaptopurine, 60 mg/m^2^, days 1–7), ML (methotrexate, 3.0 g/m^2^, day 1; L-asparaginase, 6,000 IU/m^2^, days 2 and 3) and MA (mitoxantrone, 8.0 g/m^2^, days 1–3; cytarabine, 1.5 g/m^2^, days 1–3) chemotherapies were applied for ~1 month, respectively. Prior to each consolidation regimen, a lumbar puncture combined with an intrathecal injection was performed using 10 mg methotrexate, 30 mg cytarabine and 5 mg dexamethasone. However, following intensive post-remission chemotherapy, the patient experienced bone marrow suppression with a significantly reduced total leukocyte count of 170/µl (normal range, 4,000–10,000/µl) and was affected by severe pneumonia. A 3-week course of intravenous antibiotics was administrated and the hemopoietic function of the bone marrow returned to the normal levels 1 month later. Simultaneously, molecular CR was confirmed by bone marrow examination, revealing suppression of *BCR*/*ABL* chimeric gene expression. Subsequently, the patient was subjected to consolidation treatment with the IVD regimen (imatinib, 400 mg/day; vindesine, 4 mg/day, days 1 and 11; dexamethasone, 10 mg/m^2^/day, days 1–5 and days 11–15) combined with interferon-α 2b (3 million units, twice per week) to maintain remission for 9 courses on a periodic basis (every 1 month).

Until November 2013, the patient remained asymptomatic, while sustained hematological and molecular genetic normality were achieved for ~16 months after CR. In addition, a chest CT image revealed that the bone cortex margin of the left rib was irregular and bone density was uneven in osseous thorax without evident osteolytic destruction. However, in November 2013, the patient was lost to follow-up.

The present was approved by the ethics committees of West China Hospital, Sichuan University (Chengdu, China) and written informed consent was obtained from the patient.

## Discussion

Symptoms of ALL at diagnosis usually include nonspecific manifestation of bone marrow failure, such as fever, weakness or bleeding (7). Although the presence of osteopenia/osteoporosis may be observed in both the initial and progressive phases of patients with ALL, bone pain and spontaneous bone fractures, which mainly involve the proximal limbs due to leukemic involvement along with adjacent joint pain or swelling, as the sole prodrome for ALL are rarely observed (8). As previously described, vertebral body compression fractures associated with bone pain occurred in <10% of children with ALL as the initial presentation (9). However, to the best of our knowledge, generalized osteopenia and vertebral complications presented as the only symptoms prior to the diagnosis of ALL has not been previously reported in an adult ALL patient. Based on a search of the PubMed database, to the best of our knowledge only 3 adolescent and 1 child cases have been described in the English literature to date (9–12). These cases presented with exclusive back pain and were then verified to exhibit marked osteoporosis and spontaneous vertebral compression fractures; ultimately, they were diagnosed with ALL after 3–4 months. These cases included an 8-year-old girl in Italy (10) and a 13-year-old boy in Turkey (9), as well as a 9-year-old boy and a 2-year-old boy in Bangladesh (11,12). Compression fractures of the vertebrae are known to be caused by osteoporosis (which is the most common cause), trauma to the back and tumors that develop in the bone or spread to the bone from other sites (13). A tumor that develops in the spine, including MM, is more common in older people (14). In the 56-year-old patient of the present study, a working diagnosis of MM was preliminarily established; thereby, IFE and SPE were applied to exclude MM. Notably, bone destruction is not currently the determined predictor for ALL prognosis; however, bone pain and fractures may still be major evidence to support a diagnosis of ALL and greatly influence the quality of life prior to establishing the ultimate diagnosis of ALL (15). Thus, ALL must be considered in the differential diagnosis, and close follow-up and bone marrow examination are important when a patient presents with unexplained marked osteopenia, bone pain and multiple fractures, particularly prior to establishing a definite diagnosis of idiopathic juvenile osteoporosis for children and MM for adults. In order to suppress bone resorption and reduce bone turnover, the case was treated with pamidronate disodium with no significant side-effects in the entire therapeutic course.

However, the role of imatinib in Ph^+^ ALL treatment has attracted increased attention due to the success of TKI treatment of chronic myeloid leukemia. Prior to the discovery of TKIs, Ph^+^ ALL was considered to be the high-risk group with the poorest outcome among all subtypes of ALL (16). Although a CR rate of 50–60% could be achieved by routine chemotherapy, short-term remission and high relapse rate resulted in a poor 5-year survival rate of <10% for adults and OS of only 20% (17). More intensive chemotherapy regimens were only able to improve the CR rate, rather than the long-term OS. However, with the use of TKIs in chemotherapy, the therapeutic efficacy markedly increased, reaching up to 95% in patients undergoing CR and 70% in those undergoing molecular CR, as well as resulted in improved 3-year OS of 55% (18,19). For older patients (age, ≥65 years) with Ph^+^ ALL, imatinib alone or in combination with reduced intensive chemotherapy is reported to be the first-line inductive regimen based on the National Comprehensive Cancer Network Guidelines since 2012 (4). In addition, the final results of the EWALL-Ph-01 Study were presented in 2012 at the American Society of Hematology meeting and recommended the used of the third generation TKI, dasatinib, and low intensity chemotherapy as the first-line treatment in patients with *de novo* Ph^+^ ALL aged ≥55 years (20).

The 56-year-old male patient of the present study successfully completed the IVD regimen and quickly achieved CR 2 weeks after initiation of the treatment. Whether optimal long-term efficacy may be maintained using an imatinib-based regimen, allogeneic hematopoietic stem cell transplantation (Allo-HSCT) or TKI + Allo-HSCT requires further investigation. Certain authors have recommended that TKI-based regimens may have a similar or even superior effect compared with Allo-HSCT, particularly in patients who may be at a high risk of transplantation-associated mortality, such as the elderly (21). Due to the lack of a matched donor, the current case accepted regular intensification and continued extramural imatinib as a consolidation approach. In addition, clinical studies are currently ongoing to evaluate the best procedure and duration of maintenance in patients who previously benefited from TKI-based front-line treatment. Until recently, maintenance therapy with imatinib or interferon demonstrated impressive effectiveness for the treatment of patients with Ph^+^ ALL (22).

In conclusion, based on the aforementioned evidence, the present case was subjected to interim maintenance treatment using imatinib and IFN to achieve persistent molecular CR for ~16 months. The emergence of TKIs has greatly contributed to marked improvement in the outcome of patients with Ph^+^ ALL; thus, there has been much debate regarding whether regimens including TKI could challenge the efficacy of conventional high-dose chemotherapy for such cases. To a certain extent, the present study suggests that the administration of TKIs may provide certain additional benefits for patients with Ph^+^ ALL compared with high-dose chemotherapy. For example, a common side effect of high-dose chemotherapy is poor tolerance, however, TKIs appear to be associated with improved tolerance. Further evidence and studies from additional multicenter, prospective, randomized clinical trials are required to clarify the role of reduced-intensity chemotherapy combined with imatinib for the treatment of adults with Ph^+^ ALL.
